# Dual-Phase Immunomodulation by the Bovine β-Casein Peptide KEMPFPK: Insights into Potential TLR Interaction and Gut Microbiota-Mediated Effects

**DOI:** 10.3390/foods15061080

**Published:** 2026-03-19

**Authors:** Junpeng Zhang, Xinyu Zhang, Jianping Wu, Guangqing Mu, Xiaomeng Wu

**Affiliations:** 1 School of Food Science and Technology, Dalian Polytechnic University, Dalian 116034, China; 2 Department of Agricultural, Food and Nutritional Science, University of Alberta, Edmonton, AB T6G 2P5, Canada

**Keywords:** bovine β-casein-derived peptide, immunomodulatory, NF-κB/MAPK signaling, gut microbiota, toll-like receptor

## Abstract

This study investigates the immunomodulatory effects and underlying mechanisms of KEMPFPK, a peptide derived from bovine β-casein, using integrated in vitro, in silico, and in vivo approaches. In RAW264.7 macrophages, KEMPFPK enhanced proliferation, phagocytosis, and migration and selectively upregulated the chemokine MCP-1. Under LPS-induced inflammation, KEMPFPK suppressed pro-inflammatory cytokines (IL-1β, TNF-α) and NO production while promoting the anti-inflammatory cytokine IL-10. These effects were mediated through the inhibition of NF-κB and MAPK signaling pathways. Molecular docking predicted high-affinity binding of KEMPFPK to Toll-like receptors (TLR2 and TLR4), suggesting a potential mechanism for its immunomodulatory activity. In cyclophosphamide (CTX)-induced immunosuppressed mice, KEMPFPK administration restored immune organ indices, rebalanced serum cytokine levels, and modulated humoral immunity. Importantly, KEMPFPK was associated with a significantly reshaped gut microbiota profile, characterized by the promotion of beneficial genera (e.g., *Ligilactobacillus*, *Adlercreutzia*) and the suppression of opportunistic pathogens (e.g., *Escherichia–Shigella*). These findings establish KEMPFPK as a dual-phase immunomodulator and suggest that its effects may involve direct immune cell regulation coupled with indirect microbiota remodeling. This study provides a scientific foundation for the application of KEMPFPK in immunomodulatory functional foods.

## 1. Introduction

The immune system is essential for maintaining physiological homeostasis and defending against pathogenic challenges, with its dysregulation implicated in a spectrum of diseases, including chronic inflammation, infections, and cancer [1,2]. In recent years, food-derived immunomodulatory peptides have garnered significant attention as promising natural agents for immune modulation, owing to their diverse bioactivities, such as antioxidant, antimicrobial, and immunostimulatory properties [3]. Unlike synthetic drugs, these peptides offer a safer and more sustainable alternative for supporting immune health.

Macrophages, as central players in innate immunity, are key targets for immunomodulatory interventions. Bioactive peptides derived from dietary proteins—such as those from milk, soy, and marine sources—have demonstrated the ability to fine-tune macrophage functions through multiple mechanisms, including regulation of inflammatory mediators, signaling pathways, and oxidative stress [4,5,6]. For instance, peptides from monkfish and whey protein hydrolysates have been shown to attenuate inflammation by modulating Toll-like receptor 4 (TLR4) and nuclear factor-kappa B (NF-κB) pathways while enhancing antioxidant defenses [7,8]. These effects are often mediated through critical signaling cascades, including NF-κB, mitogen-activated protein kinases (MAPKs), and the Keap1/Nrf2 pathway [6]. Moreover, the gut microbiota plays a well-established role in the regulation of host immunity, mediated in part by microbial metabolites such as short-chain fatty acids (SCFAs). For instance, SCFAs produced by Clostridium species enhance the differentiation and function of regulatory T (Treg) cells through mechanisms involving G protein-coupled receptor activation and histone deacetylase inhibition [9]. In this context, bioactive peptides have emerged as promising modulators of gut microbiota composition and immune homeostasis. They exert their effects through diverse mechanisms, including direct antimicrobial activity, immunomodulation, and metabolic intervention. Notably, certain peptides display remarkable specificity in shaping microbial communities. For example, some antimicrobial peptides (AMPs) selectively inhibit pathogens such as *Enterococcus faecium* while promoting the growth of beneficial commensals like *Faecalibacterium prausnitzii* [10,11]. Such targeted modulation can enhance intestinal barrier integrity and attenuate systemic inflammation. Despite the growing number of reports on immunomodulatory food-derived peptides, the structure–activity relationships governing their function, their direct interactions with immune receptors, and their in vivo efficacy remain poorly characterized. Furthermore, the ecological impact of peptides on gut microbial networks and the precise mechanisms underlying peptide–microbiome–immune cross-talk are still not fully understood.

In our previous work, the digestion products of β,κ-casein were obtained through simulated infant gastrointestinal digestion. Animal experiments have shown that the digests of β,κ-casein exhibited superior immunomodulatory activity [12]. Subsequent prediction of the peptides present in these digests led to the identification and selection of the KEMPFPK peptide. Building upon our previous identification of KEMPFPK, a peptide derived from bovine β-casein, this study aims to comprehensively characterize its immunomodulatory functions and explore the associated mechanisms. We employed an integrated strategy combining in vitro assays to assess macrophage functions (proliferation, phagocytosis, migration) and inflammatory responses with in silico molecular docking against key immune receptors (TLR2/TLR4) and in vivo validation in a cyclophosphamide-induced immunosuppressed mouse model. Furthermore, we investigated the impact of KEMPFPK on gut microbiota composition and dynamics. We hypothesize that KEMPFPK may exert immunomodulation through a dual interface involving direct host immune regulation and gut microbiota interaction. This work seeks to provide a multifaceted understanding of KEMPFPK’s bioactivity, laying a foundation for its potential application in functional foods aimed at immune support.

## 2. Materials and Methods

### 2.1. Materials

#### 2.1.1. Biological Materials

The murine macrophage cell line RAW 264.7 (Shanghai Cell Biology Institute, Shanghai, China) was maintained in DMEM supplemented with 10% FBS and 1% antibiotics at 37 °C under 5% CO_2_ [13]. Three-week-old male BALB/c mice were obtained from Liaoning Changsheng Biotechnology Co., Ltd. (Dalian, China).

#### 2.1.2. Chemical Materials

The bovine β-casein-derived peptide KEMPFPK (Lys-Glu-Met-Pro-Phe-Pro-Lys, molecular weight: 876.5 Da, purity: 98%) was custom-synthesized by DG Peptides Co., Ltd. (Wuhan, China). For experiments, KEMPFPK was dissolved in sterile phosphate-buffered saline (PBS) or cell culture medium at the indicated concentrations. Dulbecco’s Modified Eagle Medium (DMEM), fetal bovine serum (FBS), 0.5% Trypsin–EDTA, and penicillin/streptomycin solution were procured from Meilun Biotechnology Co., Ltd. (Shanghai, China). ELISA kits for mouse MCP-1 (Catalog Number: BPE20157), MCP-3 (Catalog Number: BPE20556), IL-1β (Catalog Number: BPE20533), IL-10 (Catalog Number: BPE20005), and TNF-α (Catalog Number: BPE20220) were obtained from Lengton Biotechnology Co., Ltd. (Shanghai, China) for detecting cell supernatants and mouse serum. Lipopolysaccharides (LPS) were supplied by Macklin Biochemical Technology Inc. Co., Ltd. (Shanghai, China). RNA extraction and qPCR kits were purchased from Seven Biotechnology Co., Ltd. (Beijing, China). The CKK-8 kit was purchased from Beyotime Biotechnology Co., Ltd. (Shanghai, China). All other general reagents were acquired from Beyotime Biotechnology Co., Ltd. (Shanghai, China).

### 2.2. Cell Culture and Immunomodulatory Assays

Cell viability was assessed using the CCK-8 assay. Cells were seeded at 5 × 10^3^ cells/well in 96-well plates, treated with KEMPFPK (25–100 μM) for 24 h, incubated with CCK-8 reagent for 2 h, and absorbance was measured at 450 nm [14].

To investigate the impact of KEMPFPK on the cell cycle, Raw 264.7 cells were incubated with or without KEMPFPK (25, 50, and 100 μM) for 24 h. Following this, the cells were fixed in cold 70% ethanol overnight and subsequently stained at room temperature for 15 min using propidium iodide staining solution. A flow cytometer was employed to analyze the samples, and FlowJo software (Version 10.8.1, BD Biosciences, Milpitas, CA, USA) was utilized to determine the percentage of cells in each phase of the cell cycle [15]. The gating strategy was detailed in the Supplementary Materials.

Cell migration was evaluated using 24‑well Transwell chambers. Conditioned media from KEMPFPK-treated cultures were placed in the lower chamber. Cells (5 × 10^5^/mL) were seeded in the upper chamber and incubated for 24 h. Migrated cells were fixed, stained with crystal violet, and counted under a microscope [16].

RAW 264.7 cells were treated with KEMPFPK for 24 h, incubated with neutral red solution for 2 h, washed, and lysed. Phagocytic activity was determined by measuring absorbance at 540 nm [17].

$$Phagocytosis\;rate\;\left(\%\right)=\frac{{OD}_{treatment}-{OD}_{blank}}{{OD}_{control}-{OD}_{blank}}\times 100$$

### 2.3. Analysis of Inflammatory Mediators and Gene Expression

To assess the immunomodulatory effects of KEMPFPK under inflammatory conditions, RAW264.7 cells were seeded in 12-well plates and pretreated with LPS (2 µg/mL) for 24 h, followed by co-incubation with the peptide (25, 50, and 100 μM) for an additional 24 h. The concentrations of TNF-α, IL-1β, IL-10, MCP-1, and MCP-3 in the culture supernatants were quantified using ELISA [18]. Nitric oxide (NO) production was determined by the Griess method, and intracellular iNOS levels were measured with a commercial ELISA kit [19]. For gene expression analysis, total RNA was extracted using a dedicated kit, reverse-transcribed into cDNA, and subjected to a StepOne Real-Time PCR System (Applied Biosystems, Foster City, CA, USA) using a Viia 7 system with gene-specific primers (Table 1) [20].

### 2.4. Molecular Docking

Molecular docking was performed to characterize the interactions between KEMPFPK and Toll-like receptors (TLRs). The crystal structures of TLR2 (PDB: 1FYW) and TLR4/MD-2 (PDB: 5IJD) were retrieved from the Protein Data Bank. Before docking, water molecules and extraneous ligands were removed from the protein structures. Docking parameters for 1FYW were set as follows: center_x = −2.699, center_y = 93.769, center_z = 22.546, size_x = 27.75, size_y = 30.0, size_z = 22.5. For 5IJD, the parameters were: center_x = −9.452, center_y = 10.855, center_z = −0.191, size_x = 39.75, size_y = 25.5, and size_z = 45.75. Docking simulations were carried out using AutoDock Vina (Version 1.2.0, The Scripps Research Institute, La Jolla, CA, USA), with the grid box parameters centered specifically on the known ligand-binding sites of each receptor. The resulting docking poses were visualized and analyzed using PyMOL (Version 1.5).

### 2.5. Immunofluorescence Staining

To evaluate the activation of the NF-κB and MAPK pathways, RAW264.7 cells grown on coverslips in 6-well plates were treated as described in Section 2.3. Following treatment, cells were washed with PBS, fixed with 4% paraformaldehyde for 15 min, and blocked for 1 h. They were then incubated overnight at 4 °C with primary antibodies against NF-κB p65, JNK, ERK, and p38. After washing, cells were incubated with an FITC-conjugated secondary antibody for 1 h, counterstained with DAPI, and mounted with an anti-fade reagent. Images were captured using an Axio Imager A2 fluorescence microscope (Carl Zeiss, Oberkochen, Germany) [21].

### 2.6. Pharmacokinetic and Tissue Distribution Analysis

All animal procedures were approved by the Animal Ethics Committee of Dalian Polytechnic University (Approval No. DLPU2024002). Three-week-old male BALB/c mice experiments are conducted with a 7-day adaptation period beforehand and housed under controlled conditions (22 ± 1 °C, 50 ± 1% humidity, 12 h light/dark cycle) with free access to food and water. All mice in this study were fed a standard commercial rodent chow (obtained from Liaoning Changsheng Biotechnology Co., Ltd.) that is free of bovine-derived proteins, including casein and its derivatives. The composition of the diet was as follows: Crude protein: 18–20% (from plant sources: soybean meal, corn gluten meal), Crude fat: 4–5%, Crude fiber: ≤5%, Ash: ≤8%, Moisture: ≤10%, Nitrogen-free extract: remaining balance.

#### 2.6.1. KEMPFPK Bioavailability Measurement

Independent cohorts of mice were used for the pharmacokinetic study. The bioavailability of KEMPFPK was evaluated in three-week-old male BALB/c mice. For the pharmacokinetic study, mice (*n* = 20) received a single intravenous injection of KEMPFPK (10 mg/kg) or oral administration of KEMPFPK (30 mg/kg). Blood samples were collected at predetermined time points (0, 2, 4, 6, 8, 10 h), and serum peptide concentrations were determined to establish time-concentration curves [22]. Serum concentrations of KEMPFPK were determined by liquid chromatography-tandem mass spectrometry (LC-MS/MS). Briefly, serum samples (50 μL) were mixed with 200 μL acetonitrile containing 0.1% formic acid for protein precipitation. After centrifugation at 12,000× *g* for 10 min at 4 °C, the supernatant was collected and analyzed using an Agilent 6460 Triple Quadrupole LC-MS system (Agilent Technologies, Waldbronn, Germany) equipped with an Agilent Eclipse Plus C18 column (2.1 × 50 mm, 1.8 μm, Agilent Technologies, Waldbronn, Germany). The mobile phase consisted of water with 0.1% formic acid (A) and acetonitrile with 0.1% formic acid (B) at a flow rate of 0.3 mL/min. The detection was performed in positive electrospray ionization mode, monitoring the transition *m*/*z* 877.5 → 438.2 for KEMPFPK. The calibration curve was linear over the range of 1–1000 ng/mL (R^2^ > 0.99), with a lower limit of quantification of 1 ng/mL. The complete pharmacokinetic parameters for KEMPFPK following intravenous and oral administration are now presented in Table 2. The relative bioavailability was calculated as (AUC_oral × Dose_iv)/(AUC_iv × Dose_oral) × 100%.

#### 2.6.2. The Tissue Distribution of KEMPFPK in Mice

Independent cohorts of mice were used for the tissue distribution study. For tissue distribution analysis, three-week-old male BALB/c mice (*n* = 15) were administered FITC-KEMPFPK (1 mg/mL) by oral gavage. At intervals ranging from 15 to 360 min post-administration, mice were euthanized, and the stomach, liver, and intestine were excised (Figure S1). The distribution of the fluorescently labeled peptide in these tissues was visualized and captured using an IVIS Lumina Series III system (Revvity, Waltham, MA, USA) [23].

### 2.7. Animal Experiments

Independent cohorts of mice were used for the immunosuppression model study. After acclimatization, three-week-old male BALB/c mice were randomly assigned to four groups (*n* = 10): Control, CTX (Model), LH (Levamisole Hydrochloride, positive control), and KEMPFPK. Immunosuppression was induced in the CTX, LH, and KEMPFPK groups via intraperitoneal injection of cyclophosphamide (80 mg/kg/bw/d) for three consecutive days, while the Control group received saline. Following modeling, the LH group was administered levamisole hydrochloride (40 mg/kg/bw), and the KEMPFPK group received KEMPFPK (100 mg/kg/bw) by oral gavage daily for 14 days. The Control and CTX groups received saline. After the treatment period, mice were euthanized. Blood was collected via orbital puncture, and serum was separated by centrifugation at 3000× *g* for 20 min. Levels of IL-1β, IL-6, IL-10, TNF-α, IgA, and IgM were measured using ELISA. The thymus and spleen were excised and weighed to calculate organ indices. For histological analysis, tissues were fixed in 4% paraformaldehyde, dehydrated, embedded in paraffin, sectioned at 5 μm thickness, and stained with hematoxylin and eosin (H&E) [24].

### 2.8. Gut Microbiota

The feces of the mice (Section 2.7) were collected after the treatment period ended. Samples were immediately frozen in liquid nitrogen and stored at −80 °C for subsequent analysis. Total genomic DNA was extracted from fecal samples using the QIAamp Fast DNA Stool Mini Kit (Qiagen, Hilden, Germany) according to the manufacturer’s instructions. The concentration and purity of the extracted DNA were assessed spectrophotometrically. The hypervariable V3–V4 region of the bacterial 16S rRNA gene was amplified using specific primers. PCR products were purified and sequenced on an Illumina MiSeq platform (Illumina, San Diego, CA, USA) according to standard protocols [12].

### 2.9. Statistical Analysis

Data analysis and graphical visualizations were performed utilizing GraphPad Prism 8.0 (GraphPad Software, San Diego, CA, USA). Results are displayed as mean values accompanied by their respective standard deviations. All experiments were independently repeated at least three times. To assess statistical significance, an initial one-way ANOVA was conducted, followed by post hoc *t*-tests, with a significance threshold established at *p* < 0.05.

## 3. Results and Discussion

### 3.1. KEMPFPK Enhances Macrophage Proliferation, Phagocytosis, and Migration

**Figure 1:**
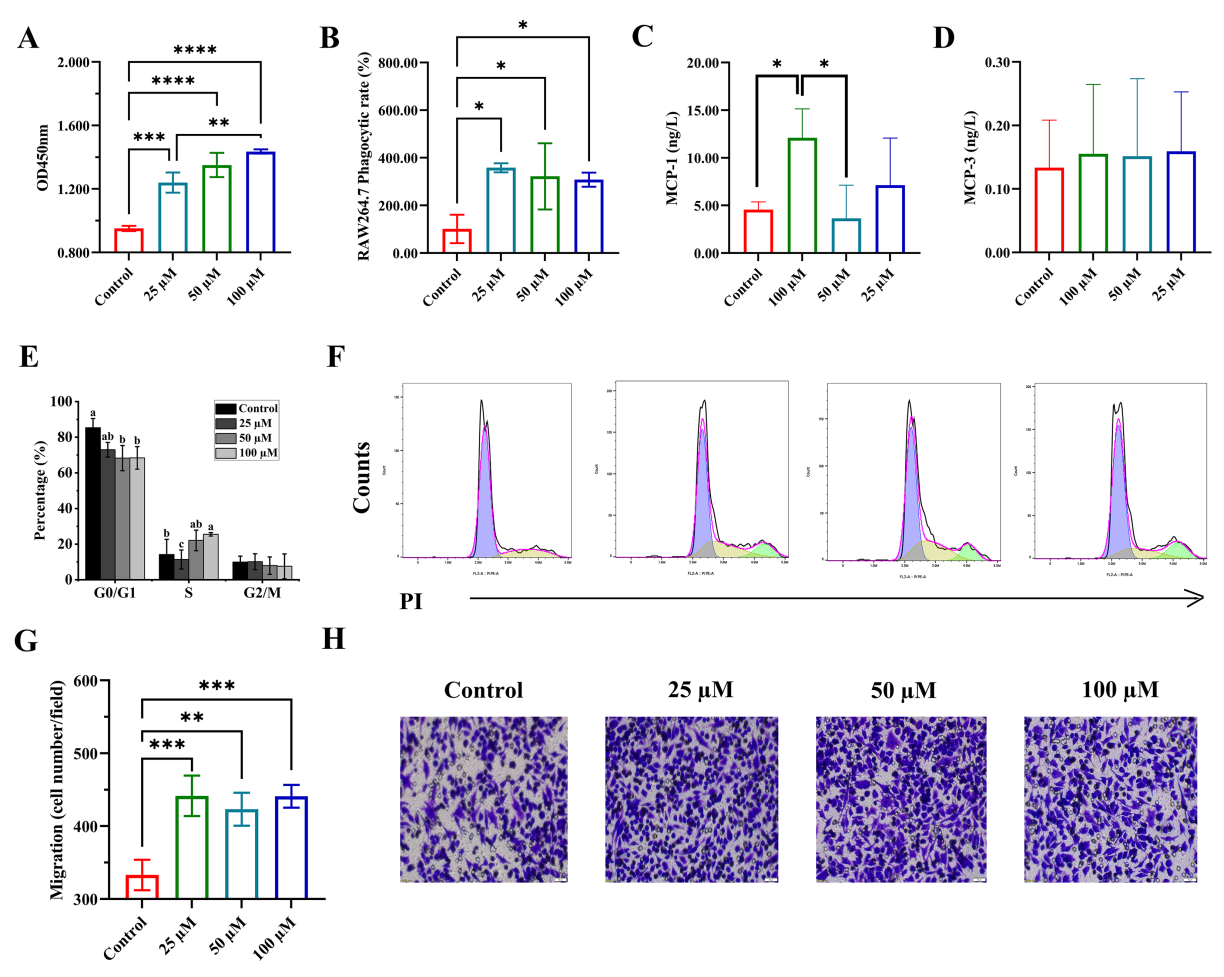
The effect of different concentrations of KEMPFPK on RAW264.7 cells. (**A**) Cell proliferation. (**B**) Phagocytic activity of RAW264.7. (**C**,**D**) The contents of MCP-1 and MCP-3 in cell supernatants. (**E**,**F**) Flow cytometry was used to analyze the cell cycle distribution of RAW264.7 cells treated with KEMPFPK. In the histogram (**E**), the purple area represents cells in the G0/G1 phase, the yellow area represents cells in the S phase, and the blue area represents cells in the G2/M phase. The percentages of cells in the G0/G1, S, and G2/M phases of the cell cycle are presented in (**E**), with a–c denoting different levels of significance. (**G**,**H**) Transwell migration assays showing comparison of migration towards medium and culture supernatants from KEMPFPK, untreated or treated Raw264.7 cells. Bar = 50 μm. Cell migration ability was calculated by counting cells per field in (**G**). * *p* < 0.05, ** *p* < 0.01, *** *p* < 0.001, **** *p* < 0.0001.

The immunomodulatory potential of the peptide KEMPFPK was first assessed by evaluating its effects on fundamental macrophage functions. As illustrated in Figure 1, KEMPFPK exerted a multifaceted, dose-dependent activation of RAW264.7 macrophages. Cell viability was significantly promoted by KEMPFPK treatment, with a detectable effect even at the lowest concentration of 25 µM (Figure 1A). This proliferative effect was mechanistically linked to the regulation of the cell cycle. Flow cytometric analysis revealed that treatment with 100 µM KEMPFPK significantly reduced the proportion of cells in the G0/G1 phase while increasing the population in the S phase (Figure 1E,F). This suggests that KEMPFPK facilitates the transition from quiescence to DNA synthesis, a process critical for immune cell expansion. In immune regulation, the proliferation of macrophages is tightly controlled, and bioactive peptides can influence these processes by modulating key signaling pathways. For instance, certain immunomodulatory peptides have been shown to enhance macrophage proliferation by increasing the tyrosine phosphorylation of MAPKs, such as ERK1/2, which are pivotal for driving cell cycle progression [25]. The observed cell cycle promotion by KEMPFPK is consistent with such a mechanism, underscoring its role as a positive regulator of macrophage expansion.

Beyond proliferation, KEMPFPK significantly enhanced the innate functional capacity of macrophages. The peptide robustly increased the pinocytic uptake of neutral red dye across all tested concentrations (25–100 µM), indicating a potent stimulation of phagocytic activity (Figure 1B). Phagocytosis is a cornerstone of innate immunity, enabling the clearance of pathogens and cellular debris. The enhancement of this function by KEMPFPK suggests its potential to bolster the first line of host defense. This effect may be mediated through the upregulation of phagocytic receptors or the activation of intracellular signaling cascades, such as the PI3K/Akt pathway and its downstream target NF-κB, which have been implicated in peptide-mediated augmentation of macrophage phagocytosis [26].

**Figure 2:**
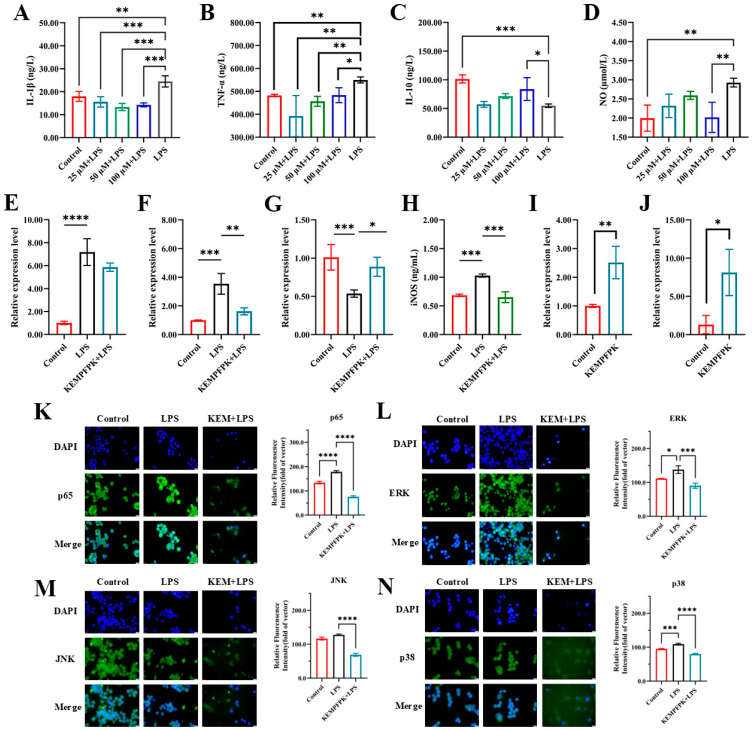
Effects of different concentrations of peptides on inflammatory factors in the mouse macrophage RAW264.7. (**A**–**D**) IL-1β, TNF-α, IL-10 and NO. (**E**–**G**) The mRNA relative expression of IL-1β, TNF-α, and IL-10. (**H**) The content of iNOS in the cell. (**I**,**J**) The mRNA relative expression of MCP-1 and MCP-3. (**K**–**N**) The effect of KEMPFPK (100 μM) on the activation of NF-κB and MAPK signaling pathways. The left panel shows the protein expression images of p65 (**K**), ERK (**M**), JNK (**L**), and p38 (**N**) observed under a fluorescence microscope; the right panel shows the immunofluorescence analysis of the protein expression levels of p65, ERK, JNK, and p38. * *p* < 0.05, ** *p* < 0.01, *** *p* < 0.001, **** *p* < 0.0001.

Furthermore, KEMPFPK demonstrated a pronounced ability to direct macrophage migration, a critical process for immune surveillance and inflammatory responses. Using a Transwell chamber assay, we found that conditioned media from KEMPFPK-treated cultures potently attracted macrophages (Figure 1G,H). This pro-migratory effect was mechanistically linked to a specific upregulation of the key chemokine MCP-1 (CCL2) (Figure 1C), with no significant impact on MCP-3 (CCL7) secretion (Figure 1D). The selective induction of MCP-1 is highly significant, as this chemokine is a principal driver of monocyte and macrophage recruitment to sites of infection or injury [27]. This finding aligns with studies on other bioactive peptides, such as RKWLRKIRRWRK, which also promote cell migration by stimulating chemokine synthesis [28]. The selective induction of MCP-1, but not MCP-3, by KEMPFPK under basal conditions suggests a specific signaling bias, potentially through differential engagement of Toll-like receptor (TLR)-mediated pathways. These receptors are known to activate distinct downstream signaling cascades, including the NF-κB and MAPK pathways, which can differentially regulate chemokine gene expression. For instance, while both pathways can contribute to MCP-1 transcription, MCP-3 expression may require additional or alternative activation signals, such as those involving IRF3 or AP-1, which may not be efficiently triggered by KEMPFPK under basal, non-inflammatory conditions. This receptor-level bias offers a plausible mechanistic explanation for the selective chemokine profile observed and aligns with the context-dependent immunomodulatory function of KEMPFPK. Notably, in the LPS-induced inflammatory context (Figure 2), KEMPFPK suppressed excessive inflammation, indicating its role is context-dependent and may avoid exacerbating damage while facilitating initial immune cell recruitment.

### 3.2. KEMPFPK Suppresses LPS-Induced Inflammation via Transcriptional and Post-Translational Regulation of Key Mediators

We next investigated KEMPFPK’s capacity to modulate the inflammatory response in an LPS-challenged environment. As depicted in Figure 2, KEMPFPK exhibited significant anti-inflammatory activity, effectively restoring cytokine balance in a dose-dependent manner. In RAW264.7 macrophages stimulated with LPS (2 μg/mL), we observed a characteristic inflammatory profile, marked by a sharp increase in the secretion of the pro-inflammatory cytokines IL-1β and TNF-α, coupled with a significant reduction in the anti-inflammatory cytokine IL-10 (Figure 2A–C). Concurrently, there was a substantial increase in nitric oxide (NO) production (Figure 2D). Treatment with KEMPFPK post-LPS challenge significantly reversed these effects. Specifically, the elevation of TNF-α was significantly suppressed at all tested concentrations (25–100 μM), while the reduction in IL-1β reached statistical significance at the higher concentrations (50 and 100 μM) (Figure 2A,B). Furthermore, KEMPFPK treatment dose-dependently restored IL-10 levels and markedly reduced NO production at the highest concentration (100 μM) (Figure 2C,D). This ability to recalibrate the cytokine milieu towards an anti-inflammatory state is a hallmark of potent immunomodulators. The suppression of NO was mechanistically linked to a downregulation of inducible nitric oxide synthase (iNOS) protein (Figure 2H), the key enzyme responsible for high-output NO generation during inflammation. This aligns with findings for other food-derived peptides, such as those from ark shell protein hydrolysate, which also exert anti-inflammatory effects by suppressing iNOS expression [29].

To determine whether this regulation occurred at the transcriptional level, we analyzed mRNA expression. The results demonstrated that KEMPFPK (100 μM) treatment significantly reduced the LPS-induced (2 μg/mL) mRNA expression of IL-1β and TNF-α while enhancing the expression of IL-10 (Figure 2E–G). This indicates that the peptide’s modulation of cytokine secretion is pre-translationally regulated. Furthermore, KEMPFPK upregulated the mRNA expression of the chemokines MCP-1 and MCP-3 (Figure 2I,J), corroborating its role in directing immune cell migration as shown in Figure 1. The coordinated downregulation of pro-inflammatory genes and upregulation of chemotactic genes underscores the sophisticated, multi-target nature of KEMPFPK’s immunomodulatory action.

The regulation of such inflammatory mediators is predominantly controlled by the NF-κB and MAPK signaling pathways. Immunofluorescence analysis revealed that LPS stimulation induced robust nuclear translocation of the NF-κB subunit p65 and activation of the MAPK components ERK, JNK, and p38 (Figure 2K–N). However, co-treatment with KEMPFPK markedly suppressed the activation of all these key signaling molecules. The phosphorylation results of p65, ERK, JNK, and p38 can further prove that KEMPFPK can inhibit the activation of these pathways, but this requires further research. The NF-κB and MAPK pathways are central hubs in inflammation, and their coordinated activation by LPS leads to the transcription of pro-inflammatory genes, including TNF-α, IL-1β, and iNOS. The ability of KEMPFPK to concurrently inhibit both pathways provides a compelling mechanistic explanation for its broad-spectrum anti-inflammatory efficacy. This mode of action is shared by other natural anti-inflammatory compounds, such as magnoflorine and asiaticoside, which also suppress inflammation by dual inhibition of NF-κB and MAPK phosphorylation [30,31]. Our findings position KEMPFPK within this class of bioactive food components that function by targeting master regulators of inflammation.

### 3.3. Molecular Docking

**Figure 3:**
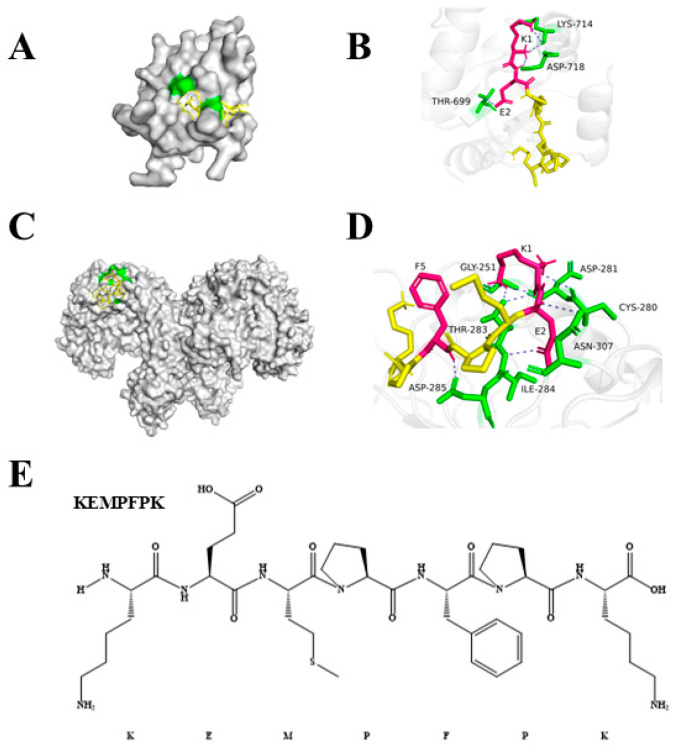
Molecular docking result of KEMPFPK with TLR2 and TLR4/MyDD. (**A**,**B**) With TLR2. (**C**,**D**) With TLR4/MyDD. (**E**) KEMPFPK structure.

To explore a potential molecular initiation event, we performed molecular docking simulations to investigate its interaction with key immune receptors, specifically Toll-like receptors 2 and 4 (TLR2 and TLR4). The results demonstrated that KEMPFPK binds strongly to both TLR2 and TLR4/MyDD complexes, with calculated binding affinities of −7.5 kcal/mol and −6.0 kcal/mol, respectively (Table 3). These values indicate predicted high-affinity interactions, comparable to or stronger than those reported for some known immunomodulatory peptides interacting with TLRs. They are consistent with reported binding energies for known immunomodulatory peptides, which typically exceed −3.5 kcal/mol [32]. A detailed analysis of the binding interfaces revealed that KEMPFPK forms multiple specific hydrogen bonds with critical residues in the TLR binding pockets. As shown in Figure 3, KEMPFPK forms four hydrogen bonds with TLR2, involving residues THR699, ASP718, and LYS714. Notably, the glutamate (E) and lysine (K) residues within the KEMPFPK sequence played a pivotal role in these interactions, contributing to the stability of the peptide-receptor complex. Similarly, in the TLR4/MyDD complex, KEMPFPK formed six hydrogen bonds with residues ASP285, THR283, ASN307, ASP281, and CYS280. The central involvement of Glu and Lys residues in binding to both TLRs provides a structural rationale for the observed immunomodulatory effects of KEMPFPK. These findings align with previous studies on bioactive peptides from food sources. For instance, immunomodulatory peptides from *Litopenaeus vannamei* heads, such as PSPFPYFT and GPQGPPGH, also rely heavily on Glu and Lys residues for stabilizing interactions with TLR2 and TLR4/MyDD [33]. Similarly, donkey-hide gelatin-derived peptides VQLSGEEK and GFSGLDGAKG demonstrate high TLR4-MD-2 affinity through critical hydrogen bonds formed by Lys residues with key receptor residues [34]. The strong binding affinity and specific interaction pattern observed in our docking studies suggest that KEMPFPK could potentially modulate immune responses by, or in part by, interacting with TLR2 and TLR4, potentially acting as a ligand or modulator for these receptors. This molecular recognition event provides a plausible mechanism for the downstream effects observed in our cellular studies, including the inhibition of NF-κB and MAPK signaling pathways and the subsequent modulation of inflammatory cytokine production. TLR4, in particular, is a well-known receptor for LPS-induced inflammatory signaling, and the binding of KEMPFPK to the TLR4/MyDD complex may interfere with LPS binding or receptor dimerization, thereby exerting an anti-inflammatory effect. While the docking predictions are supportive, functional validation using TLR-specific reporter assays will be necessary in future work to confirm whether KEMPFPK acts as an agonist, antagonist, or allosteric modulator of these receptors.

### 3.4. Pharmacokinetics and Tissue Distribution of KEMPFPK in Mice

**Figure 4:**
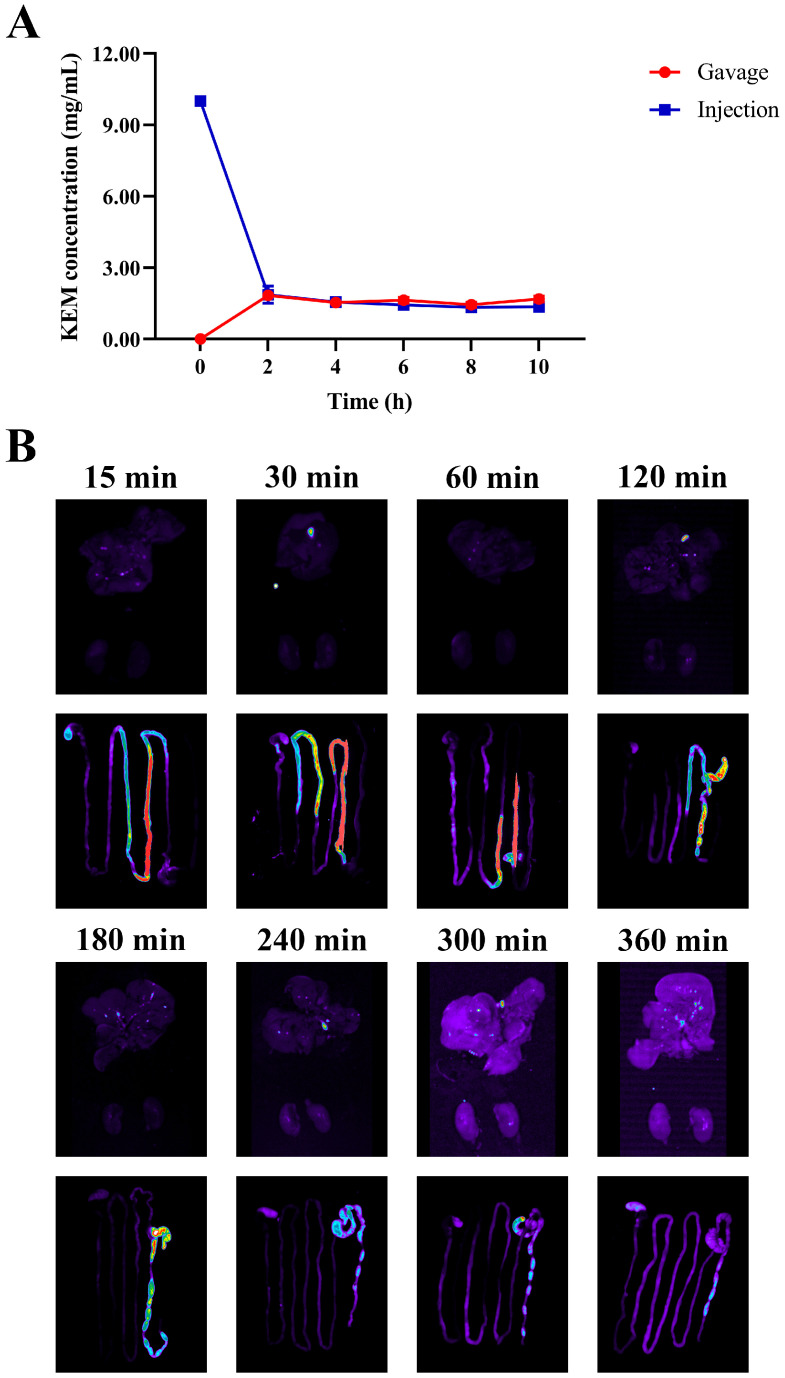
Effects in serum and tissues of mice after administration of KEMPFPK. (**A**) Time-concentration curves of peptide concentrations in the serum of mice after intravenous injection of KEMPFPK (10 mg/kg) or oral administration of KEMPFPK (30 mg/kg). (**B**) Fluorescence images of the stomach, liver, and spleen of mice after intragastric administration of FITC-KEMPFPK for 15–360 min.

The pharmacokinetic profile and tissue distribution of KEMPFPK were investigated to evaluate its bioavailability and targeting potential in vivo. The doses used in the pharmacokinetic study were selected based on preliminary toxicity and efficacy studies, as well as references from similar bioactive peptide studies. The intravenous dose of 10 mg/kg was chosen to ensure detectable systemic exposure without causing adverse effects, while the oral dose of 30 mg/kg was selected to reflect a typical therapeutic range for food-derived peptides in murine models. These doses allowed for a reliable comparison of bioavailability and tissue distribution. As shown in Figure 4A, following intravenous administration (10 mg/kg), KEMPFPK exhibited rapid clearance from the bloodstream, with serum concentrations declining sharply within the first 60 min. In contrast, oral administration (30 mg/kg) resulted in a delayed but sustained absorption phase, with detectable peptide levels persisting for up to 360 min. After calculation, the relative bioavailability of KEMPFPK was found to be 6.133%. Tissue distribution studies using FITC-labeled KEMPFPK (Figure 4B) revealed rapid accumulation in key immune organs. Following oral administration of KEMPFPK-FITC, strong fluorescence was observed in the digestive organs within 15–120 min, indicating rapid transit and initial interaction with the gastrointestinal tract. Notably, fluorescence was detected in the liver as early as 30 min and persisted at low levels for up to 6 h, suggesting partial systemic absorption. After 120 min, significant fluorescence was localized predominantly in the large intestine (specifically the cecum and colon), where it remained detectable for several hours before declining, with only faint signals in the colon by 6 h. This spatiotemporal pattern implies that a portion of KEMPFPK is absorbed systemically, while a substantial fraction reaches the lower gut, where it may be utilized by the intestinal microbiota or undergo excretion. These observations align with the relative bioavailability, indicating low systemic exposure but significant retention and potential activity within the gut environment. The persistent presence of the peptide in colonic regions supports the hypothesis that KEMPFPK may exert immunomodulatory effects not only through direct absorption and systemic action but also via modulation of the gut microbiota, which in turn influences host immunity. This dual-pathway mechanism is consistent with reports on other food-derived peptides, such as those from soybean and marine sources, which have been shown to enhance immune function both directly through interactions with Toll-like receptors and subsequent NF-κB/MAPK signaling and indirectly by promoting beneficial microbial communities and reinforcing gut barrier integrity [33,35]. The low systemic bioavailability yet significant in vivo efficacy suggests that the local actions of KEMPFPK in the gastrointestinal tract—including potential direct effects on gut-associated lymphoid tissue and the observed remodeling of the gut microbiota—may play a predominant role in its systemic immunomodulatory outcomes, with limited absorbed peptide contributing to effects on peripheral immune organs.

### 3.5. KEMPFPK Ameliorates Cyclophosphamide-Induced Immunosuppression and Modulates Inflammatory Homeostasis In Vivo

**Figure 5:**
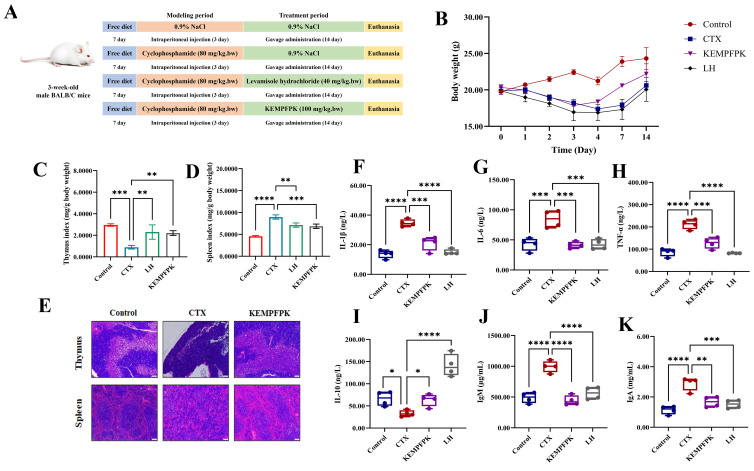
Effect of peptide KEMPFPK on immunosuppressed mice. (**A**) Animal experimental design; (**B**) body weight of mice at different time points; (**C**) thymus index; (**D**) spleen index; (**E**) mouse thymus and spleen staining (scale bar: 200); (**F**–**K**) mice serum levels of IL-1β, IL-6, TNF-α, IL-10, IgM, and IgA. * *p* < 0.05, ** *p* < 0.01, *** *p* < 0.001, **** *p* < 0.0001.

To validate the immunomodulatory efficacy of KEMPFPK in a physiological context, we employed a cyclophosphamide (CTX)-induced immunosuppression model in mice. The results presented in Figure 5 demonstrate that KEMPFPK administration significantly counteracted CTX-induced immune dysfunction, restoring immune organ integrity and rebalancing systemic inflammatory mediators. CTX treatment led to a marked reduction in body weight (Figure 5B) and severe atrophy of primary and secondary immune organs, as evidenced by decreased thymus and spleen indices (Figure 5C,D). Histopathological examination further confirmed this damage, revealing a thinner thymic medulla with lymphocyte loss and disorganized splenic architecture with blurred red-white pulp boundaries (Figure 5E). Oral administration of KEMPFPK (100 mg/kg/bw) for 14 days substantially mitigated these effects. The peptide treatment facilitated faster body weight recovery, significantly improved thymus and spleen indices, and restored the histological architecture of both organs, characterized by lymphocyte repopulation and clearer delineation of splenic zones (Figure 5C–E). The protection of immune organs from CTX-induced damage is a critical indicator of immunorestorative capacity. Our findings align with studies on other food-derived peptides, such as those from shrimp hydrolysate, which have been shown to modulate immune responses in immunosuppressed models by protecting lymphoid tissues [36]. At the systemic level, CTX challenge triggered a pronounced inflammatory state, characterized by elevated serum levels of the pro-inflammatory cytokines IL-1β, IL-6, and TNF-α, along with a decrease in the anti-inflammatory cytokine IL-10 (Figure 5F–I). KEMPFPK treatment effectively reversed this imbalance, significantly suppressing the pro-inflammatory cytokines while boosting IL-10 production. This cytokine rebalancing effect underscores the peptide’s ability to fine-tune the immune response, corroborating our in vitro findings in macrophages. Furthermore, KEMPFPK administration modulated humoral immunity, as indicated by the normalization of serum levels of IgA and IgM, which were elevated in the CTX model group (Figure 5I,K). This reduction towards control levels suggests that KEMPFPK helped restore immune homeostasis rather than causing generalized immunosuppression. Similar immunomodulatory effects have been observed for egg white peptides, which were shown to ameliorate immune imbalance in immunosuppressed mice [37]. In the immune disorder model induced by CTX, humoral immunity may simultaneously exhibit both inhibition and abnormal activation. The KEMPFPK treatment restores the elevated serum levels of IgA and IgM to normal ranges, which may reflect its overall regulatory effect on immune homeostasis rather than merely immunosuppression. The comprehensive in vivo efficacy of KEMPFPK can be mechanistically linked to its multi-target action observed in cellular models. The peptide’s ability to rebalance cytokine production likely stems from its capacity to inhibit the NF-κB and MAPK signaling pathways, as demonstrated in RAW264.7 cells. Moreover, its direct interaction with TLR2 and TLR4, revealed by molecular docking, provides a plausible initial trigger for this immunomodulatory cascade in vivo. The resulting anti-inflammatory environment, coupled with the direct protective effects on immune organs, positions KEMPFPK as a potent natural immunomodulator. In conclusion, our animal study provides compelling evidence that the bovine β-casein-derived peptide KEMPFPK effectively alleviates CTX-induced immunosuppression. It operates through a multifaceted mechanism involving the protection of immune organs, restoration of cytokine balance, and modulation of humoral immunity.

### 3.6. KEMPFPK Modulates Gut Microbiota Composition in Immunosuppressed Mice

**Figure 6:**
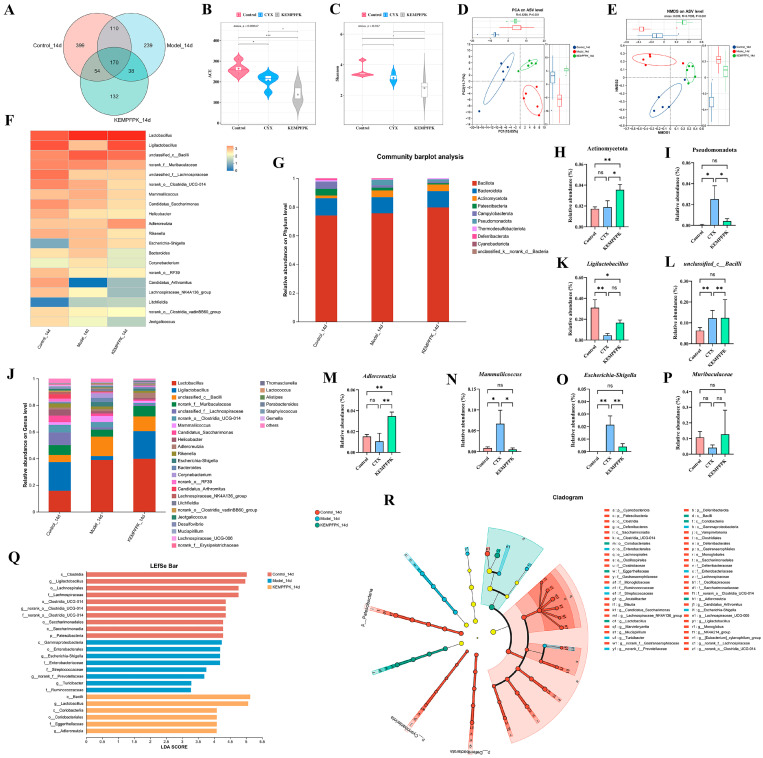
Effects of KEMPFPK on gut microbiota in mice by cyclophosphamide. (**A**) Venn diagram at the OTU level. (**B**) ACE index at the OTU level. (**C**) Shannon index at the OTU level. (**D**) PCA based on the relative abundance of gut microbiota at the OTU level. (**E**) NMSD is based on the relative abundance of gut microbiota at the OTU level. (**F**) Heatmap of gut microbiota differences at the genus level. (**G**) Relative abundance of gut microbiota at the phylum level. (**H**,**I**) Relative abundance of Actinomycetota and Pseudomonadota. (**J**) Relative abundance of gut microbiota at the genus level. (**K**–**P**) Relative abundance of *Ligilactobacillus, unclassified_c_Bacilli, Adlercreutzia, Mammaliicoccus, Escherchia-Shigella,* and *Muribaculaceae*. (**Q**) LDA scores histogram of significantly bacterial taxa (class, order, family, and genus) among the Control, CTX, and KEMPFPK groups, LDA score > 3. (**R**) LefSe analysis taxonomic cladogram. ^ns^ *p* > 0.05, * *p* < 0.05, ** *p* < 0.01, *** *p* < 0.001.

The impact of KEMPFPK on gut microbiota composition was systematically evaluated in cyclophosphamide (CTX)-induced immunosuppressed mice. As shown in Figure 6A, 16S rRNA sequencing revealed that the number of intestinal OTUs in the control group, CTX group, and KEMPFPK group was 733, 557, and 394, respectively. Among them, there were 170 common OTUs, and the corresponding unique OTU numbers were 399, 239, and 132, respectively (Figure 6A). KEMPFPK reduced ACE and Shannon indices compared to the CTX group (*p* < 0.05, Figure 6B,C). These findings indicate that there are differences in the composition of the intestinal microbiota among the various groups. This may also reflect a selective enrichment of specific beneficial taxa (e.g., *Ligilactobacillus*) during the restoration process from a severely dysbiotic state. Whether this represents a transient state or a stable new equilibrium requires longer-term study. However, beta-diversity analysis via PCA and NMDS showed clear separation between the CTX and KEMPFPK groups, with the latter clustering closer to the healthy control, suggesting a restorative effect on microbial community structure (Figure 6D,E). To further clarify the differences in the structure of the intestinal microbiota, we conducted a cluster analysis on the intestinal microbiota of mice (Figure 6F). The results showed that there were differences among the three groups in *Lactobacillus*, *Ligilactobacillus, unclassified_c__Bacilli, norank_f__Muribaculaceae, unclassified_f__Lachnospiraceae, norank_o__Clostridia_UCG-014, Mammaliicoccus, Candidatus_Saccharimonas, Helicobacter, Adlercreutzia, Rikenella, Escherichia–Shigella, Bacteroides, Corynebacterium, norank_o__RF39, Candidatus_Arthromitus, Lachnospiraceae_NK4A136_group, Litchfieldia, norank_o__Clostridia_vadinBB60_group,* and *Jeotgalicoccus*. At the phylum level, KEMPFPK intervention counteracted the CTX-induced depletion of Firmicutes and expansion of Bacteroidota, resulting in a significantly improved Firmicutes/Bacteroidota ratio (*p* < 0.01) while also enhancing the abundance of Actinomycota and reducing the abundance of Pseudomonadota compared to the CTX group (Figure 6G–I). The Actinomycota includes many probiotics, such as *Bifidobacterium*, whose increased abundance is often closely associated with the maintenance of immune homeostasis. *Bifidobacteria* can regulate the differentiation of regulatory T cells (Treg), inhibiting excessive inflammatory responses and enhancing intestinal barrier function [38]. This suggested that KEMPFPK might promote the growth of beneficial bacteria in the intestines of mice, thereby increasing the abundance of beneficial bacteria. Pseudomonadota include various opportunistic pathogens such as *Escherichia coli* and *Pseudomonas aeruginosa*. Their excessive proliferation can activate the Toll-like receptor 4 (TLR4) signaling pathway through pathogen-associated molecular patterns like lipopolysaccharides (LPS), promoting the release of pro-inflammatory factors (IL-6, TNF-α) and exacerbating systemic inflammation [39,40,41]. This indicated that KEMPFPK could inhibit the growth of harmful bacteria induced by cyclophosphamide. Most notably, genus-level analysis demonstrated that KEMPFPK specifically enriched beneficial commensals, including *Ligilactobacillus* and *Adlercreutzia*, while simultaneously suppressing opportunistic pathogens such as *Escherichia–Shigella* and *Mammaliicoccus* (Figure 6K–P). Existing research indicates that *Ligilactobacillus* can activate Th1-type immune responses, promoting the secretion of IFN-γ and IL-2, thereby enhancing cellular immune defense capabilities [42,43]. For instance, *L. salivarius* CCFM 1266 enhances the host’s resistance to bacterial and fungal infections by upregulating the expression of IL-17 [42], while *L. murinus* alleviates inflammatory responses in atherosclerosis by inhibiting the expression of the key protein GSDMD involved in macrophage pyroptosis [44]. The immunomodulatory effects of *Adlercreutzia* were primarily achieved through its metabolic products. This genus can metabolize isoflavones to generate anti-inflammatory active products [45], and its increased abundance is significantly correlated with a reduction in systemic inflammatory markers such as CRP [46]. In an alcoholic liver disease (ALD) model, the symbiotic network of *Adlercreutzia* and *Lachnospiraceae* inhibits the NF-κB pathway by activating the GPR109A receptor, thereby alleviating liver inflammation [44,47]. The overgrowth of *Escherichia–Shigella* is often associated with a pro-inflammatory state, and its enrichment in immunocompromised individuals may exacerbate intestinal barrier dysfunction and systemic inflammatory responses [48,49]. Additionally, *Mammaliicoccus* may increase the risk of nosocomial infections through horizontal gene transfer, while virulence factors like β-lactamase can trigger excessive inflammatory responses mediated by neutrophils [49]. This indicates that KEMPFPK can regulate the growth of beneficial bacteria and inhibit the reproduction of harmful bacteria. Furthermore, LEfSe also analysis identified *Ligilactobacillus, Bacilli*, and *Lactobacillales* as the most differentially abundant taxa in the KEMPFPK group (LDA score > 4.0), confirming the peptide’s selective promotion of immunomodulatory microorganisms (Figure 6O–R). The restoration of gut microbiota structure, especially the enrichment of *Ligilactobacillus* and *Adlercreutzia*, indicates that it may be correlated with systemic immune homeostasis.

### 3.7. KEMPFPK Modulates Specific Gut Microbial Taxa in Immunosuppressed Mice

**Figure 7:**
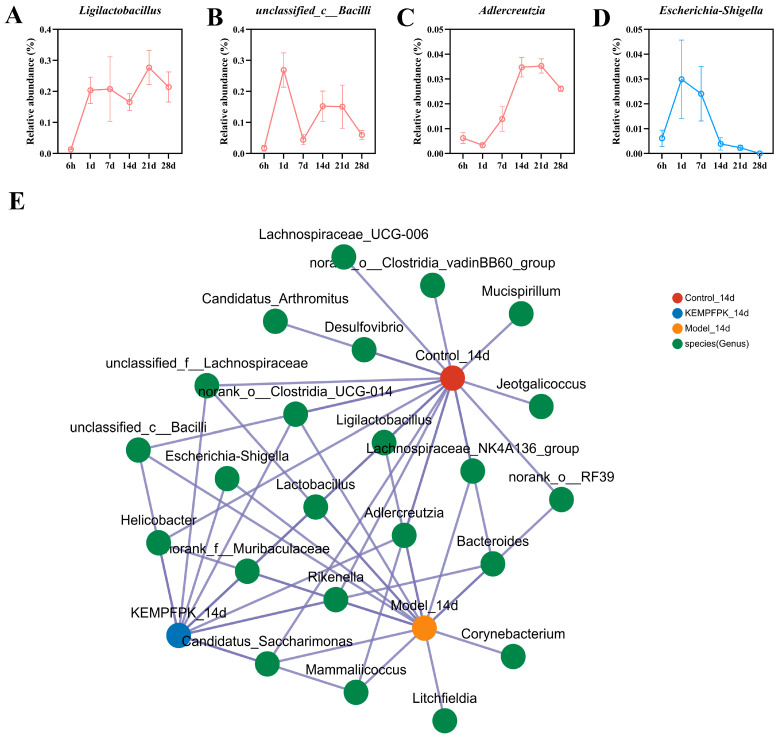
Effects of KEMPFPK on gut microbiota in mice by cyclophosphamide. (**A**–**D**) The changes in *Ligilactobacillus, unclassified_c_Bacilli, Adlercreutzia,* and *Escherichia–Shigella* in mice by cyclophosphamide after the KEMPFPK intervention. (**E**) Collinearity analysis plot of gut microbiota at the genus level.

Analysis of temporal changes in specific bacterial taxa following KEMPFPK intervention revealed distinct colonization patterns and ecological succession in CTX-treated mice (Figure 7). The beneficial genus *Ligilactobacillus* exhibited a progressive increase in relative abundance throughout the intervention period, suggesting successful growth promotion by KEMPFPK (Figure 7A). Similarly, *Adlercreutzia*, known for its anti-inflammatory properties through equol production, showed sustained enrichment over time, indicating stable establishment of this health-associated taxon (Figure 7C). In contrast, the potentially pathogenic *Escherichia–Shigella* demonstrated a rapid decline upon KEMPFPK administration, with suppression maintained consistently during the treatment course (Figure 7D). Interestingly, we observed that the *unclassified_c__Bacilli* also gradually decreased over a long period of KEMPFPK intervention. However, much research indicates that *Bacilli* can modulate host immune responses through various pathways. For instance, strains such as *Bacillus subtilis* and *Bacillus amyloliquefaciens* can significantly enhance intestinal barrier function and inhibit the release of pro-inflammatory factors (e.g., TNF-α and IL-6), thereby alleviating systemic inflammatory responses [50,51]. Certain Bacillus strains may produce toxins or trigger excessive immune activation under specific conditions [52]. For example, in patients with COPD, dysbiosis of the gut microbiota leading to the overgrowth of *Bacteroides fragilis* can compromise the intestinal barrier, triggering systemic inflammation and exacerbating pulmonary lesions [50]. We hypothesized that specific suppression of harmful Bacilli may be beneficial. The collinearity analysis further revealed evolving microbial correlations during intervention, with strengthened positive associations between beneficial taxa and negative correlations with pathogens in the KEMPFPK group (Figure 7E). Notably, the network in KEMPFPK-treated mice demonstrated strengthened alliances between *Ligilactobacillus* and other commensals, forming cooperative clusters that likely contribute to community stability and functional redundancy. Concurrently, the pathogenic genus *Escherichia–Shigella* occupied a marginalized position in the network, exhibiting negative correlations with multiple beneficial taxa, suggesting effective competitive exclusion. This network reorganization reflects a fundamental restoration of microbial social architecture, where KEMPFPK promotes the formation of a resilient, mutually supportive bacterial community. We speculate that the changes in the bacterial community structure might be related to the systemic immune regulation of KEMPFPK. However, this study did not conduct fecal microbiota transplantation (FMT) or quantitative analysis of metabolites (such as short-chain fatty acids), so a direct causal relationship cannot be established. This correlation provides a clear direction for future in-depth exploration of its mechanism.

An important consideration for the translational potential of KEMPFPK is its potential antigenicity and risk of inducing hypersensitivity reactions. As a peptide derived from bovine β-casein, a common food allergen, KEMPFPK theoretically carries a risk of eliciting immune responses in susceptible individuals, particularly those with cow’s milk protein allergy. However, KEMPFPK is a heptapeptide (7 amino acids), which is below the typical threshold (8–9 amino acids) required for MHC class II presentation and T-cell activation, potentially reducing its immunogenicity [53]. When administered orally, as in our study, the peptide is biased towards tolerance induction rather than active immunity [54]. In addition, no adjuvants were used in any of our administrations, further reducing the risk of inducing a robust adaptive immune response. Furthermore, numerous studies have demonstrated the safety of food-derived peptides in both animal models and human trials, with rare reports of hypersensitivity [55]. Nevertheless, we acknowledge that a comprehensive safety evaluation, including allergenicity assessment and repeated-dose toxicity studies, would be necessary before any clinical application. Future studies should investigate potential IgE cross-reactivity with existing milk allergens and evaluate the immune response following long-term administration. These considerations are essential for the responsible development of KEMPFPK as a functional food ingredient or therapeutic agent.

## 4. Conclusions

This study provides comprehensive evidence that the bovine β-casein-derived peptide KEMPFPK possesses potent and multifaceted immunomodulatory activities. Our integrated approach demonstrates that KEMPFPK enhances fundamental macrophage functions (proliferation, phagocytosis, and migration) and exhibits anti-inflammatory properties in vitro, which are associated with the suppression of the NF-κB and MAPK signaling pathways. Molecular docking analysis predicted high-affinity binding of KEMPFPK to the key immune receptors TLR2 and TLR4, offering a plausible structural basis for its observed cellular effects. In a cyclophosphamide-induced murine model of immunosuppression, KEMPFPK administration effectively restored immune organ indices, rebalanced systemic cytokine levels, and modulated humoral immunity. Concurrently, KEMPFPK intervention was associated with a significant and dynamic remodeling of the gut microbiota, characterized by the selective promotion of beneficial genera (e.g., *Ligilactobacillus*, *Adlercreutzia*) and suppression of opportunistic pathogens (e.g., *Escherichia–Shigella*). Collectively, these findings position KEMPFPK as a promising dual-phase immunomodulator. Its effects may stem from a combination of direct host immune regulation and indirect, microbiota-associated modulation. This work establishes a foundational rationale for further exploration of KEMPFPK as a bioactive component in functional foods designed to support immune homeostasis.

## Figures and Tables

**Table 1:** Genes and Corresponding Primers Used in RT-qPCR.

Gene	Primer	Sequence (5′ to 3′)	Annealing Temperature (°C)
*MCP-1*	Forward	GGCTCAGCCAGATGCAGTTAA	60
	Reverse	CCTACTCATTGGGATCATCTTGCT	60
*MCP-3*	Forward	AAGAAGGGCATGGAAGTCTG	58
	Reverse	TCAAGGCTTTGGAGTTGGG	58
*TNF-α*	Forward	CTGGATGTCAATCAACAATGGGA	60
	Reverse	ACTAGGGTGTGAGTGTTTTCTGT	60
*IL-1β*	Forward	TGTGAAATGCCACCTTTTGA	58
	Reverse	TGAGTGATACTGCCTGCCTG	58
*IL-10*	Forward	CAGAGCCACATGCTCCTAGA	60
	Reverse	TGTCCAGCTGGTCCTTTGTT	60
*GAPDH*	Forward	AGGTCGGTGTGAACGGATTTG	60
	Reverse	GCAGCTCTAGGAGCATGTGG	60

**Table 2:** Pharmacokinetic parameters of KEMPFPK in mice.

Parameter	Unit	IV (10 mg/kg)	Oral (30 mg/kg)
C_max_	ng/mL	1250 ± 85	78.5 ± 6.2
T_max_	min	5	60
T½ (elimination half-life)	min	45.3 ± 4.2	112.6 ± 8.7
AUC_0–480_	min·ng/mL	28,450 ± 1250	5230 ± 280
AUC_0–∞_	min·ng/mL	29,680 ± 1380	5460 ± 310
MRT (mean residence time)	min	52.4 ± 3.8	168.5 ± 12.4
CL (clearance)	mL/min/kg	0.34 ± 0.03	-
Vd (volume of distribution)	mL/kg	22.1 ± 1.8	-
Relative bioavailability (F)	%	-	6.13

**Table 3:** Molecular Docking of Peptides with Toll Receptors.

Sequence	Affinity (kcal/mol) TLR2	Affinity (kcal/mol) TLR4/MyDD
KEMPFPK	−7.5	−6

## Data Availability

The original contributions presented in the study are included in the article/Supplementary Materials; further inquiries can be directed to the corresponding author.

## Supplementary Materials

The following supporting information can be downloaded at https://www.mdpi.com/article/10.3390/foods15061080/s1, Figure S1: Animal experiment design. (A) represents the pharmacokinetic animal experiment design for KEMPFPK. (B) represents the animal experiment design for the tissue distribution of KEMPFPK in mice. Figure S2: Gating strategy for cell cycle analysis by flow cytometry.
