# Supplementary material for: Dual-Phase Immunomodulation by the Bovine β-Casein Peptide KEMPFPK: Insights into Potential TLR Interaction and Gut Microbiota-Mediated Effects
Source: Foods. 2026 Mar 19;15(6):1080. doi: 10.3390/foods15061080 (PMC13025458; doi:10.3390/foods15061080)
Supplement: Supplementary file 1 [file foods-15-01080-s001.zip › foods-4144552-supplementary.pdf]

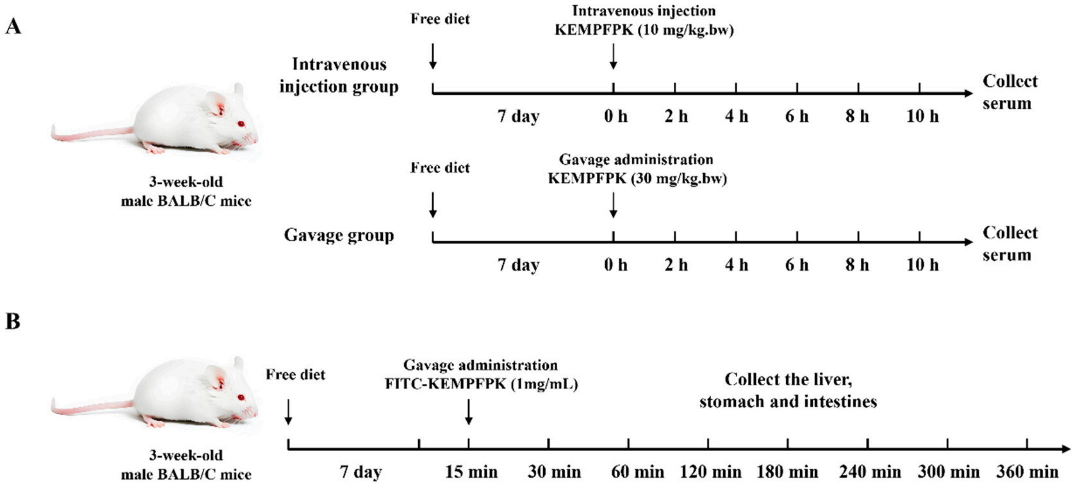

Figure S1 Animal experiment design. (A) represents the pharmacokinetic animal experiment design for KEMPFPK. (B) represents the animal experiment design for the tissue distribution of KEMPFPK in mice.

Gating strategy for cell cycle analysis by flow cytometry.

First, cell debris and dead cells were excluded using the FSC-A/SSC-A scatter plot. Subsequently, doublets were removed through the FSC-A/FSC-H scatter plot to ensure the analysis was performed on single cells. Finally, the G0/G1, S, and G2/M phases were fitted and analyzed using (BD Biosciences, USA) in the PI channel histogram. A representative gating strategy is illustrated in Figure S2. Figure S2 take the Control group as an example.

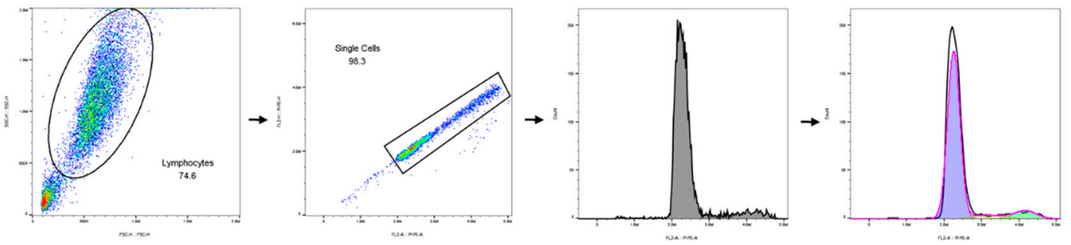

Exclusion of cell debris and dead cells using FSC-A/SSC-A scatter plot.

FSC-A/FSC-H scatter plot for doublet discrimination.

PI channel histogram.

FlowJo software fitting analysis.

Figure S2 Gating strategy for cell cycle analysis by flow cytometry.
